# COVID-19 and its threat to refugees in Africa

**DOI:** 10.34172/hpp.2021.33

**Published:** 2021-08-18

**Authors:** Emery Manirambona, Laura Wilkins, Don Eliseo Lucero-Prisno III

**Affiliations:** ^1^College of Medicine and Health Sciences, University of Rwanda, Kigali, Rwanda; ^2^Medical Sciences Division, University of Oxford, Oxford, United Kingdom; ^3^Department of Global Health and Development, London School of Hygiene and Tropical Medicine, London, United Kingdom; ^4^Faculty of Management and Development Studies, University of the Philippines (Open University), Los Baños, Laguna, Philippines

**Keywords:** Africa, COVID-19, Healthcare disparities, Refugees, SARS-CoV-2

## Abstract

Although it is widely accepted that coronavirus disease 2019 (COVID-19) has adversely affected the Global South’s most vulnerable refugee communities, they have received little attention. There have been gaps in testing, which is fundamental to treat and isolate patients and make data-driven decisions to protect the refugee community. Therefore, it is imperative to holistically implement policies to curtail COVID-19 in refugee camps to ensure that refugees are safe and protected from the pandemic. Processes for timely diagnosis and treatment, quick isolation and contact tracing are essential to keep refugees safe. Furthermore, it is crucial to encourage protective behaviours and raise awareness about hygiene and social prevention to dampen disease transmission. Refugees in the Global South have been disproportionately affected by the consequences of the COVID-19 pandemic, facing financial hardship and social injustice throughout. Refugees in Africa have also faced threats to their security, being subjected to torture, disappearance, or even killings in their host countries. The pandemic has exposed gender inequalities, with females being the most affected, and health inequities in the refugee community in Africa. There is a need for international organizations like the African Union, United Nations (UN) agencies, non-governmental organizations (NGOs), and other stakeholders to take serious action regarding the refugee situation in Africa. Food aid for refugees in Africa should be increased as quickly as possible and refugees’ security must be guaranteed. Of equal importance, there must be justice for the death or disappearance of refugees. It is imperative to end discrimination against refugees and support the promotion of gender equity.

## Background

Ever since coronavirus disease 2019 (COVID-19) was declared a pandemic by the World Health Organization on 11 March 2020, Africa has been severely hit. All countries on the African continent have been affected, and the situation continues to evolve. As of 23 June 2021, Africa had registered 5 330 048 COVID-19 cases^1^ and confirmed 139 506 deaths.^2^ Yet, many cases have gone unreported as gaps in testing have been evident in some countries.

Despite Africa’s quick actions to curtail the spread of COVID-19, the economies of most African countries have been significantly affected. For instance, the World Bank estimated the African economy to have declined by 3.3% in 2020, marking ‘the region’s first recession in 25 years’.^3^ The financial impact of the COVID-19 pandemic on African low- and middle-income countries has been unavoidable and is even more evident among refugee communities in Africa.

Before the onset of the COVID-19 pandemic, African health systems and security were already vulnerable. Lack of essential aspects that strengthen the health system, such as good health governance and leadership, health surveillance and appropriate health workforce, impended health security.^4^ Moreover, food insecurity and hunger prevailed for multiple reasons such as escalating armed conflicts and socio-economic grievances.^5^ Some East African countries suffer significantly from locust invasions, while other pests affect the Sahel region and southern Africa. West Africa also suffered food insecurity due to crop yield reduction following poor rainfall, whereas floods ravaged some eastern African countries.^6^ The Global Hunger Index score of many African countries is classified as either alarming or serious (35-49.9 and 20-34.9 on a 100-point scale, respectively).^5^ COVID-19 has exacerbated these issues, with refugees suffering the most.

Although it is widely accepted that COVID-19 has disproportionately affected Africa’s most vulnerable refugee communities, they have received little attention.^6^ In this paper, we discuss the challenges faced by African refugees due to the COVID-19 pandemic and the consequences thereof, thus providing evidence for regional and global policymakers to take action on refugee issues and foster sustainable solutions.

## The impact of COVID-19 on refugees in the Global South

Although there was a higher predilection of COVID-19 cases and mortality rates among refugees and internally displaced camps compared to other groups, the toll has been lower than expected. As of February 2021, nearly 50 000 cases of COVID-19 and approximately 450 deaths had been reported among the global population of 80 million refugees and displaced people.^7^ The reported number of COVID-19 cases and deaths is also low among refugee camps in the Global South. It is clear that these low numbers are related to limited testing capacity in refugee settings, where the healthcare system is vulnerable. For instance, the United Nations High Commissioner for Refugees (UNHCR) reported 104 COVID-19-positive cases among refugee returnees from Tanzania to Burundi at the border in control of international travel.^8^ It is noteworthy that those refugees were formerly thought to be uninfected. Such figures indicate the low testing capacity and the possible transmission rate of the pandemic in hundreds of refugee camps in Africa. Notably, Uganda, a host of the most significant number of refugees in Africa of around one million, has tested 8219 refugees, amongst whom 503 were confirmed positive with nine deaths.^9^ This shows refugees were infected where testing was carried out.

Even though the numbers of COVID-19 cases in the refugee community in Africa are not well known, the consequences of COVID-19 are indisputable and pernicious.

Refugees in African countries have struggled to cope with the crisis caused by COVID-19, with consequences for their health, wellbeing, food supply and security. Refugees residing in many African countries have seen their food rations almost halved because international donations received by the UN World Food Programme (WFP) have decreased.^10,11^ Although governments distributed food to their citizens during lockdowns, refugees were intentionally excluded.^12^ This is in addition to worsening food shortages and rising prices in the African market due to lockdown measures, further endangering refugees’ already precarious food supply.^13^

The health and wellbeing of refugees in the Global South have deteriorated, with COVID-19 and lockdowns exacerbating their poor living conditions. First, most refugee camps are densely populated with limited or no access to essentials such as water and soap, increasing susceptibility to communicable diseases such as COVID-19.^14^ Secondly, refugees are not fed adequately. Consequently, they are at risk of hunger, poor growth, malnutrition and anaemia.^15^ Under these circumstances, the COVID-19 pandemic has increased their disease vulnerability.

Social injustices have occurred during the implementation of preventive measures in some African areas.^16^ Lockdown restrictions fuelled refugees to stay isolated indoors to avoid stigma, negatively affecting the mental health of this at-risk population.^16^

Mental health disorders are prevalent amongst refugees who have experienced trauma due to conflicts and war in their countries of origin.^17^ Of note, refugees depending entirely on food rations given by the WFP are particularly likely to have a poor mental health status. As they live in uncertain and hopeless conditions, it is apparent that they are at higher risk of mental health disorders like depression and anxiety, which aggravates pre-existing post-traumatic stress disorder.^18^

While refugees seek protection in host countries, many are not allowed to register during the COVID-19 pandemic and some face nefarious actions. A large number of refugees in countries such as Tanzania are killed, tortured, go missing or are forced to return to their countries.^19,20^ Kenya also threatened to shut down its two largest refugee camps, affecting around 400 000 refugees.^21^

Refugees in Africa are at risk of forced return to their countries of origin. They face destitution and discrimination in their host countries, refugee camps are being forcibly closed, and many refugees are forced to leave; thus, they are left with no other option than to risk their lives upon return. This has implications not only for the refugees themselves but also for their countries of origin. While refugees who are not ready to return still face the fear of torture, killing, persecution and arbitrary arrest exacerbated by discrimination, their countries still experience political, racial, and ethnic tensions. Most importantly, their countries are not prepared or willing to receive them. Forced return not only exposes refugees to avoidable risks but also violates the right to free movement and financial opportunities of Africans in Africa. Additionally, it generates anti-refugee sentiments amongst the locals. The COVID-19 outbreak worsened the inequality between female and male refugees and disproportionately affected female refugees, the world’s most vulnerable category, as they are at risk of sexual harassment, sexual exploitation, sexual and gender-based violence, trafficking, teenage pregnancy and domestic violence.^22^ Notably, food shortages have forced some female refugees to exchange sex for their survival, putting them at further risk of sexually transmitted diseases. Equally important, COVID-19 restrictions led to the closure of sexual and reproductive healthcare (SRH) services and significantly disrupted the contraceptive supply chain.^12^ Female refugees have therefore been left without access to essential care such as contraceptives and other SRH services. While formal businesses selling goods continued running in South Africa, female refugees surviving by informally selling goods on the street were prohibited or arrested without support, further driving them into hardship.^23^ COVID-19 also significantly affected the education of young refugees, which disproportionately affected young girls.^24^ Female refugees were more likely to lose their jobs because of COVID-19 lockdown policies, and young girls were less likely to enrol in education.^24^

## Conclusion

Refugees in the Global South have been disproportionately affected by the COVID-19 pandemic. Although there are not many documented cases of confirmed disease and mortality in refugee camps, it is clear that this low number is correlated to a lack of testing laboratories in refugee settings. The healthcare system is vulnerable without equipped facilities and adequate healthcare staff. Even though the numbers of COVID-19 cases are not well known in the refugee community in Africa, the consequences of the pandemic are indisputable and pernicious. Refugees have faced financial hardship, social injustice, and threats to their security in their African host countries. COVID-19 has also further exposed gender inequality and health inequity in African refugee communities.

## Implication for practice and policy

The new wave of COVID-19, which continues to soar in Africa, could primarily affect refugees. It is imperative to devise holistic safeguarding measures to curb COVID-19 in refugee camps to ensure refugees are safe and protected from the pandemic. Processes for timely diagnosis and treatment, quick isolation and contact tracing are essential to keep refugees safe. Further, it is crucial to encourage protective behaviour and raise awareness about hygiene and social prevention measures to dampen disease transmission. In this regard, essentials including water and soap must be made freely available to refugees. Likewise, it is crucial to give refugees access to COVID-19 vaccines, which is the only way to end the COVID-19 pandemic.

As COVID-19 incidence is increasingly reported in Africa, we advocate for additional health facilities, equipment, drugs and PPE in refugee camps alongside enough trained health personnel to boost their knowledge of managing infections and outbreaks. The healthcare system should ensure that healthcare services are available on an ongoing basis and that no disease is neglected to avoid the complication of existing conditions, mainly non-communicable diseases. Furthermore, considering the significance of mental health disorders and their exacerbation by the COVID-19 pandemic, psychological support should be provided to prevent the worsening of refugees’ mental health status.

Regarding the other issues revealed in the COVID-19 era, there is an urgent need for country leaders, the African Union, United Nations (UN) agencies, non-governmental organisations and other stakeholders to acknowledge the severity of the refugee situation in Africa. Action aiming to increase food aid to refugees starving in African countries is urgently needed. Additionally, the security of refugees and provision of justice to those who went through discrimination, died or disappeared should be prioritised. Refugees and their families should be central in national and regional discussions. Gender equality and humanism in refugee camps in Africa should be promoted. In order to ensure effective and equitable responses to refugee needs, the UNHCR, WFP and other stakeholders must involve refugees in decision-making locally, regionally and globally.

## Acknowledgements

The authors appreciate the reviewers for their insightful comments. The authors claim that no part of this paper is copied from other sources.

## Funding

Not applicable.

## Competing interests

None.

## Ethical approval

Not applicable.

## Authors’ contributions

EM conceptualized and designed the paper and prepared the original draft. LW and DELP assisted with data interpretation, edited, and critically reviewed the paper for intellectual content. All authors approved the final version.
